# Palm Date Fibers: Analysis and Enzymatic Hydrolysis

**DOI:** 10.3390/ijms11114285

**Published:** 2010-11-01

**Authors:** Marzieh Shafiei, Keikhosro Karimi, Mohammad J. Taherzadeh

**Affiliations:** 1 Department of Engineering, University of Borås, Borås, Sweden; E-Mails: X080298@utb.hb.se (M.S.); Mohammad.Taherzadeh@hb.se (M.J.T.); 2 Department of Chemical Engineering, Isfahan University of Technology, Isfahan, 84156-83111, Iran

**Keywords:** palm date, fiber, enzymatic hydrolysis, settling, date composition

## Abstract

Waste palm dates were subjected to analysis for composition and enzymatic hydrolysis of their flesh fibers. The fruit contained 32% glucose and 30% fructose, while the water-insoluble fibers of its flesh consisted of 49.9% lignin and 20.9% polysaccharides. Water-insoluble fibers were settled to 55% of its initial volume in 12 h. The presence of skin and flesh colloidal fibers results in high viscosity and clogging problems during industrial processes. The settling velocity of the fibers was improved by enzymatic hydrolysis. Hydrolysis resulted in 84.3% conversion of the cellulosic part of the fibers as well as reducing the settling time to 10 minutes and the final settled volume to 4% of the initial volume. It implies easier separation of the fibers and facilitates fermentation processes in the corresponding industries. Two kinds of high- and low-lignin fibers were identified from the water-insoluble fibers. The high-lignin fibers (75% lignin) settled easily, while the low-lignin fibers (41.4% lignin) formed a slurry suspension which settled very slowly. The hydrophilicity of these low-lignin fibers is the major challenge of the industrial processes.

## 1. Introduction

Date palm (*Phoenix dactylifera* L.) is widely planted in hot and dry climate regions of Africa, the Middle East and Asia. Date palm fruit is an important food resource in these regions. Annual production of dates was about seven million tons in 2004. Besides food-grade date production, large amounts of dates end up as waste. The global waste palm date production is approximately two million tons per year [1]. Waste date has harder texture and more fibers than the commercial edible grade fruit. However, it is an excellent source of sugar to produce refined sugar, concentrated juice, and confectionary pastes as well as fermentation products [2,3]. Palm date fruits consist of three main parts: date flesh, date pit, and skin. Date pit is mainly used as animal feed. The main sugars of date flesh are glucose, fructose and sucrose. At early stages of maturing the fruit, it has a high content of sucrose, but during the maturation process it is converted to glucose and fructose. Proteins appear in date fruits as 1–3% of dry matter, while its fat content was reported to be 0.52–3.25% [1,4–10]. Date skin is a thin layer surrounding the fruit to protect the fleshy part. Fat was reported to be one of the components playing a protective role in the skin [4]. However, far too little attention has been paid to the skin composition.

Fibers are the solid insoluble part of date flesh, mainly composed of cellulose, hemicellulose, lignin, and insoluble proteins. The amount of these fibers is higher in early stages of fruit life. However, during the ripening process, cellulase and pectinase enzymes present in the fruit break down insoluble polymers to smaller soluble molecules. The amount of crude fiber in commercial fruits is about 2–6%, while it could be up to 10% for low-quality date fruits [4]. These fibers can be used as dietary fibers due to oil and water uptake, and swelling capacity [8]. Palm date and its fibers have several nutritional values such as antioxidant activity [5,11]. The antioxidant activity could be due to its lignin content, since lignin has been shown to possess antioxidant and antimicrobial activities [12]. However, the presence of fibrous particles in the date flesh results in cloudy syrup. A clarification process is necessary to obtain high-quality jam and syrups.

Non-clarified date juice can be fermented in order to produce ethanol, organic acids, wine, and single-cell protein [4]. However, the presence of skin and flesh colloidal fibers results in high viscosity and clogging problems during fermentation and especially distillation processes. Therefore, it is necessary to eliminate skin and fiber particles in the syrup to overcome these problems. There are two previous reports on the effect of cellulase and pectinase enzymes on sugar extraction and syrup properties from palm dates [7,13]. However, no previous study on enzymatic hydrolysis of date fibers was detected. Furthermore, the efficient hydrolysis of cellulosic materials needs a pretreatment prior to enzymatic hydrolysis, which is a costly process and consumes energy and chemicals [14]. However, there is no report on the necessity of the pretreatment before enzymatic hydrolysis of the fibers in date palm.

The aim of this work was to determine the composition of palm date, with a specific look at skin and flesh fibers. Furthermore, study of enzymatic hydrolysis of flesh fibers by application of different preparations of cellulase, hemicellulase and β-glucosidase enzymes was carried out, and their effect on settling of fibers as a means of fiber separation was studied.

## 2. Material and Methods

### 2.1. Characterization of Palm Date Fruits

Waste palm date fruit of the Dalaki variety (Khoozestan, Iran) was used in this work. Date palm fruit life has several stages: Kimri, Khalaal, Rutab, and mature Tamr. During these stages, water content of the fruit is reduced from 85% (at the early Kimri stage) to about 20% at Tamr stage [4]. The fruit which was used in this work was in Tamr stage of maturity and rather stiff, due to its low moisture content. The three main parts of the fruit (flesh, pit and skin) were separated and subjected to experiments individually. The proportion of each part, as well as percentage of dry content in each part, was determined according to the method developed by Ehrman [15].

In order to determine date flesh composition, its sugars were first extracted. Sugar extraction yield of the date flesh depends on the temperature of the water and time of extraction, as well as mixing and particle size of the fruit. Meanwhile, reduction of free sugars in the insoluble part of the flesh is also desirable. Therefore, the following method was developed to obtain the best extraction result. Palm dates (200 g) were manually pitted. The fleshy part was ground and mixed with 1000 mL of water for 20 min at room temperature, and the remaining fibers were separated from the syrup by vacuum filtration. The fibers were then mixed with 700 mL water, and their size was reduced using a homogenizer. The suspension was then heated for 1 h at 100 °C, followed by centrifugation at 10,000 × g for 10 min. The centrifuged fibers were washed with water three times to remove the remaining soluble sugars and then freeze-dried. The resulting fibers were a mixture of all flesh and skin fibers, and hereafter are called “palm date fibers”.

Palm date fibers are composed of two kinds of fibers, one with high lignin content (dark color) and the other with low lignin content (white color). It is possible to separate these two fibers by repeated centrifugation and separation of non-dried date fibers. The low-lignin fibers have lower specific gravity and their particles are harder to settle, while the high-lignin fibers settle much faster.

### 2.2. Enzymatic Hydrolysis of Solid Part

Enzymatic hydrolysis of the palm date fibers was carried out using cellulase from *Trichoderma reesei* (80 FPU/mL activity, SIGMA C2730), β-glucosidase (240 IU/mL activity, SIGMA G0395) and hemicellulase (1.5 U/mg activity, SIGMA) according to a previous procedure [14,16]. Two different cellulase loading were examined; 20 FPU per gram of solid fibers, which is a moderate cellulase loading, and 50 FPU/gram solid, which is considered as a high enzyme loading in hydrolysis of cellulose. Effects of addition of zero or 50 IU β-glucosidase and zero or 20 U hemicellulase per gram solid fibers were also examined. The enzymes were added to 80 mL buffer solution containing 4% fibers at pH 5.0, in aseptic conditions, and hydrolysed for 48 h at 45 °C.

### 2.3. Analysis

The carbohydrates, ash, acid-soluble and insoluble lignin of flesh, low-lignin, high-lignin and skin fibers were analysed according to the methods for lignocelluloses previously published by Ruiz and Ehrman [17], Templeton and Ehrman [18], and Ehrman [19]. The compositions of the syrup and hydrolysates were analysed using HPLC (Waters, Milford, USA) by an ion-exchange column (HPX-87P, Bio-Rad, USA). Dry weight of initial and final solids matters from each flask was determined according to the procedure previously described by Ehrman [15].

The functional groups in low-lignin, high-lignin and date palm fibers as well as the hydrolysed fibers (with 50 FPU/g cellulase, 50 IU/g β-glucosidase and 20 U/g hemicellulase) were examined using a Fourier transform infrared (FTIR) spectrometer (Impact 410, Nicolet Q5 Instrument Corp., WI, USA). The spectra were obtained with an average of 60 scans and a resolution of 4 cm^−1^, in the range of 600 to 4,000 cm^−1^ [20], controlled by Nicolet OMNIC 4.1 analyzing software. The intensity of spectra was divided into corresponding intensity values to get normalized data from 0 to 1.

### 2.4. Settling

Wet date fibers were subjected to enzymatic hydrolysis with the same conditions as described above. Settling of date fibers before and after hydrolysis was investigated. The settling was performed in 100 mL measuring cylinders. Hydrolysed samples were washed and centrifuged twice. The pellet was suspended in Milli-Q water to give a final volume of 100 mL in a measuring cylinder, and they were well mixed before settling. The supernatant was taken out after 10 min and poured into another centrifuge tube. The supernatant and settled fibers were then centrifuged again and dried by freeze drier. Settling velocity was determined by measurement of settled fiber average movement divided by time. Unhydrolysed fibers had no significant settling velocity in the 100 mL measuring cylinder; therefore it was diluted 2.5 times and settling was measured in a 250 mL cylinder.

## 3. Results

### 3.1. Chemical Composition of Palm Date Fruit

The composition of the waste palm dates was analysed and the structural contents of flesh, pit and skin are presented in Table 1. The major part of the palm date fruit was the flesh with 87.6% of wet weight, while date seed and skin were about 12.0% and 0.4% of palm date fruit fresh weight. Date flesh was further analysed for moisture content, water-soluble content and water-insoluble materials. Glucose and fructose were the main constituents of date flesh with 32.0% and 30.0% of the fruit wet weight (Table 1). However no sucrose was detected. Treating the flesh with hot water resulted in extracting 97.7% of these sugars to the syrup, while the rest of the sugars remained in the water-insoluble fibers (Table 2). This insoluble material of the flesh consisted of 49.9% lignin, of which 47.7% was acid-insoluble lignin (Table 2). Other components such as galacturonic acid and acetic acid were detected, but in trace amounts (data not shown). Considering mass balance during the extraction process, negligible amounts of loss were detected, indicating negligible amounts of oligomers present in the date flesh.

The composition of the low- and high-lignin fibers separated from palm date fibers is presented in Table 2. High-lignin fibers contained 75% lignin and 15.8% polysaccharide, while the low-lignin fibers had 27.2% lignin and 53.1% polysaccharide. Palm date fiber consists of 54% high-lignin fiber and 46% low-lignin fiber. A minor part of the free sugars in the date flesh remained in the skin, but they composed only 3.9% of the skin. The other components of the skin were 40.8% polysaccharide and 41.4% lignin (Table 2). The hemicellulose of the fibers contained glucan, xylan, galactan, mannan and arabinan. The xylan and galactan are the dominant carbohydrate of the fibers, thus the fibers are similar to hardwoods and straws, rather than softwoods which contain high amount of mannan. Other components were ash, protein, and extractives.

**Figure 1.**
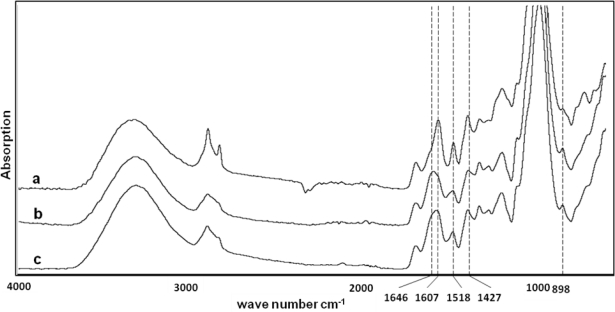
FTIR spectra of high lignin fiber (**a**) and low lignin fiber (**b**) and the date palm fiber (**c**).

FTIR was applied to examine the structure of low- and high-lignin fibers and the spectra are shown in Figure 1. The 1,427 and 898 cm^−1^ absorption bands, which are assigned to the crystalline cellulose I and cellulose II, respectively, were used to study the type of crystalline cellulose. The absorbance ratio A_1,427_/A_898_ or total crystallinity index (TCI) was calculated for the fibers [21]. The TCI of high-lignin and low-lignin fiber were 0.85 and 0.71, respectively, indicating presence of more crystalline cellulose in high-lignin fiber (Table 3). The absorption bands at 1607 cm^−1^ and 1518 cm^−1^, assigned to the stretching of the aromatic ring (lignin), were used to study lignin in the fibers, while absorption at 1634 cm^−1^ and 1646 cm^−1^ is assigned to cellulose and hemicellulose, respectively [22]. The wide band between 1607 cm^−1^ and 1646 cm^−1^ for low-lignin fiber indicates presence of cellulose and hemicellulose, while for high-lignin fibers the band is sharp at 1607 cm^−1^ and shows less cellulose and hemicellulose (Figure 1 and Table 3).

### 3.2. Enzymatic Hydrolysis

**Figure 2.**
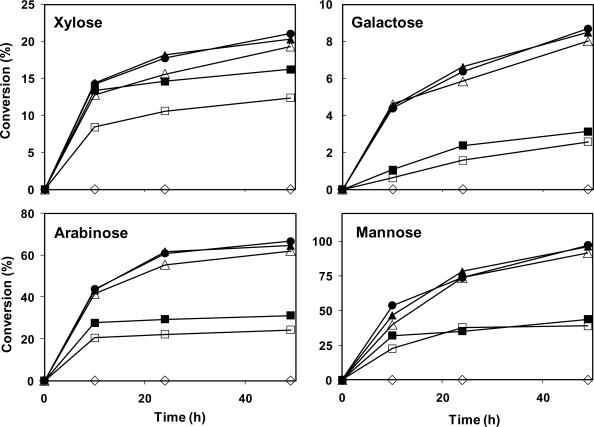
Effect of cellulase, β-glucosidase and hemicellulase on hemicellulose of flesh solid. Data is presented as yield of hydrolysis: gram of converted polymeric sugar to gram of initial hemicellulose. The presented results are averages of the two replicates. Average standard deviation is 3.1%, 3.8%, 1.1% and 0.8% for xylose, galactose, arabinose and mannose, respectively. Figure legend: (□) Cellulase 20 FPU; (▪) Cellulase 50 FPU; (Δ) Cellulase 20 FPU, β-glucosidase 50 IU; (▴) Cellulase 50 FPU, β-glucosidase 50 IU; (•) Cellulase 50 FPU, β-glucosidase 50 IU, hemicellulase 20 U; (⋄) Hemicellulase 20 U.

The enzymatic hydrolysis of date fibers (mixture of flesh and skin fibers) was carried out by using cellulase, hemicellulase, pectinase and/or β-glucosidase, and the results were examined for glucose, fructose, cellobiose, xylose, galactose, mannose, arabinose and galacturonic acid. The summary of the results is presented in Table 4 and Figure 2. The hydrolysis of the fibers with pectinase enzyme had no significant effect on fibers, and only negligible amounts of galacturonic acid were detected (data not shown). Therefore, this enzyme was not used in the subsequent hydrolyses.

Enzymatic hydrolysis with only hemicellulase enzyme had no effect on glucan or other polysaccharides in the flesh fibers (Table 4). Cellulase with 20 and 50 FPU/g activity was able to hydrolyse 37.5 and 67.1% of the glucan in the fibers, respectively (Table 4). Addition of β-glucosidase resulted in improving the hydrolysis of the fibers to 81–82%, while this yield was improved to 84.3% by the addition of hemicellulase (Table 4).

Presence of other sugars in the hydrolysates indicates hydrolysis of hemicellulose to its monomer sugars (Figure 2). Fructose concentration was constant in all the samples, regardless of the enzyme used (data not shown), probably indicating that fructose only remained from incomplete sugar extraction and there is no polymeric fructose in the sample. The best results for hydrolysis yields of xylose, galactose, arabinose, and mannose (from the hemicellulose) were 21.1%, 8.7%, 66.7%, and 96.9%, respectively, when using 50 FPU/g cellulase, 50 IU/g β-glucosidase and 20 U/g hemicellulase enzymes. The overall yield of hemicellulose hydrolysis was 31.0%. Furthermore, it was not possible to measure the oligomers resulting from incomplete hydrolysis of polysaccharides. However, the oligomer contents can be calculated from the mass balance data. When the initial weight of samples was subtracted from the produced sugars as well as remaining solids, 10.1–16.3% weight losses were observed, which is most likely the oligomer contents of the hydrolysate.

Total crystallinity index (TCI) calculated for the fibers showed 39% increase from 0.79 to 1.1 in crystallinity of the remaining fibers after hydrolysis (Table 3). An absorption band at 1518 cm^−1^ showed the presence of more lignin in the fibers after hydrolysis. The wide band between 1607 cm^−1^ and 1646 cm^−1^ for palm date fiber indicated presence of cellulose and hemicellulose, while for the hydrolysed fiber the band was sharp at 1607 cm^−1^ and showed less cellulose and hemicellulose (data not shown). However, the intensity of the absorption band at 1646 cm^−1^ for the hydrolysed sample had a higher value (0.219) than the fibers before hydrolysis (0.188), representing presence of more cellulose and hemicellulose in the hydrolysed fibers. A possible reason for this contrast was an increase in baseline at 1646 cm^−1^ because of high amounts of intensive lignin absorption at 1607 cm^−1^.

### 3.3. Settling

**Figure 3.**
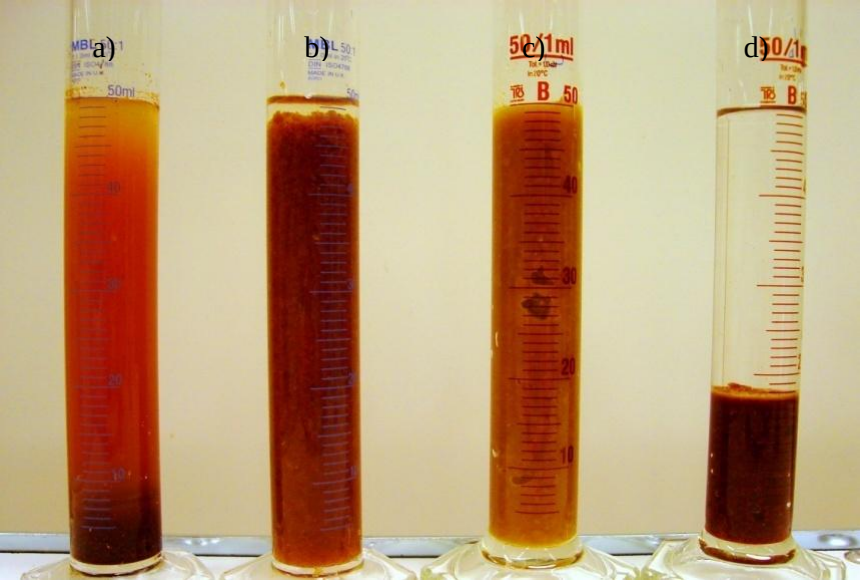
(**a**) hydrolysed fiber, (**b**) un-hydrolysed fiber, (**c**) low lignin fiber, (**d**) high lignin fiber. The amounts of dry matters in all cylinders were 2 g. The hydrolysed fibers (**a**) settles very fast and the supernatant was a cloudy liquid. Unhydrolysed fiber (**b**) settled to final volume of about 4 g/100 mL and low lignin content fibers (**c**) never settled. However, the high lignin content fibers (**d**) settled very fast and supernatant was clear water.

Unhydrolysed fibers settled very slowly by shrinking of the whole fiber layer, and the upper layer in the measuring cylinder was clear water. The average settling velocity for unhydrolysed fiber was 0.34 mL/min in the first hour, but reduced to about 0.1 mL/min in 12 h. The unhydrolysed fibers settled to 55% of their 250 mL volume in 12 h, and the final volume for the unhydrolysed solid was about 100 mL. However, the hydrolysed fibers were not in slurry form anymore and a growing layer of sedimented particles formed at the bottom of the measuring cylinder, while the upper layer was a cloudy suspension of very small particles (Figure 3). More than 80% of the particles present in all of the hydrolysed samples were settled to an approximate volume of less than 10 mL (out of 100 mL total volume) by 10 minutes. Therefore the average settling velocity for hydrolysed fiber was 3.6 mL/min, considering that only 80% of the fiber mass was settled. The total dry weight of the hydrolysed fibers was reduced to about half the amount of the unhydrolysed fibers because of removal of polysaccharides and extractive substances.

It was mentioned previously that two kinds of fiber were separated from date fibers. Figure 3 shows palm dates fibers before and after hydrolysis, as well as low- and high-lignin fibers. The amount of fiber in all measuring cylinders is 40 g/L. The high-lignin fibers settled in less than one minute in a 50 mL measuring cylinder, while the same amount of low-lignin fibers had a very viscous suspension which was not settled. This latter slurry was so thick that even air bubbles were trapped inside the suspension. Hydrolysis of these two kinds of fibers improved the settling velocity, especially for the low-lignin fibers. For high-lignin fibers, the settling volume was reduced, whereas the low-lignin fibers changed completely from thick slurry to a cloudy suspension and some fast-settling particles.

## 4. Discussion

Waste palm date is produced in large quantities in the world [1]. Appreciable amounts of free hexoses sugars are available in the palm date. However, fibers in the palm make some clogging problems in the corresponding processes, such as concentrated syrup, date honey, vinegar, ethanol, and wine production. In these processes, one has to separate the fibers using expensive equipment and loses part of the valuable sugars, which remain in the separated fibers.

The results show that 62% of wet weight of the tested palm dates was glucose and fructose sugars. Since this low-quality date is not a food-grade product, the high-sugar-content waste can be used as raw material for many biological products such as ethanol. Seeds of the dates are usually easy to separate, but the fibers present in the flesh can lead to a clogging problem in industrial processes. On the other hand, separation of the fibers with centrifugation or filtration needs expensive equipment. Settling is an inexpensive process of solid separation compared to other methods, since it does not need any energy requirements or expensive equipment e.g. centrifuge. However, a part of the sugars remain in the wet separated fibers, which typically results in the loss of 5–10% of the sugars in palm date. This problem can be solved using cellulase and hemicellulase enzymes. The enzymes increase the settling velocity of fibers by liquefaction of the polysaccharides and elimination of colloidal particles. Therefore the remaining particles of this enzymatic hydrolysis settle much faster (more than 26 times) than unhydrolysed fiber, while the final settled volume is reduced to 4% of the initial volume of unhydrolysed fiber.

Ethanol produced from glucose has a theoretical yield of 0.51 g/g, while the practical yield is typically about 0.45 g/g. It is, therefore, possible to produce about 428 liters of ethanol per ton from the sugars in palm date (dry basis), if the fiber is separated to avoid the practical problems. However, by hydrolyzing the fibers, the ethanol production yield would increase to about 454 liters, which is about 5.6% increase in the production as well as solving fiber problems.

Cellulose and hemicellulose have more hydrophilic groups such as hydroxyl groups, while lignin has a phenol ring which is hydrophobic. A possible reason for increasing settling velocity of the hydrolysed sample is the reduction of hydrophilic groups and side chains of polysaccharides present in the fibers, giving less colloidal properties. This reason could be applied to the slow settling velocity of low-lignin fibers and fast settling of high-lignin fibers.

Successful conversion of the cellulose part with 84.3% yield indicated that no pretreatment is needed for hydrolysis of palm date flesh solid. Hemicellulose could be hydrolysed up to 31.0%, although as a result of cellulase enzyme and hemicellulase enzyme together. Lignin is present in palm date fiber to about 50%. This percentage is higher than that of usual lignocellulosic materials, such as wood, which is about 30% [23]. It is reported that, during the ripening process, cellulase and pectinase enzymes break down fiber to smaller molecules [10]. This could be a reason for the higher amount of lignin. However, lignin could inhibit enzymatic hydrolysis of cellulose by adsorption of enzyme on its surface, instead of enzyme adsorption by the cellulose [24].

## 5. Conclusions

The results showed that these waste or low-quality dates contain 62% sugars, which corresponds to 77% of their dry weight. Hydrolysis of the fibers present in this material makes it a very attractive resource for biological processes. Successful hydrolysis and removal of these fibers (up to 84.3% of cellulose and 31% of hemicellulose) resulted in much faster (about 26 times) settling of the remaining particles, thus making them easier to separate, and a better quality of date syrup and fewer problems in fermentation processes.

## Figures and Tables

**Table 1.** Date palm fruit main parts.

**Part**	**Content (% dry basis)**

Seed	13.8 ± 0.29
Flesh including:	85.7 ± 0.12
Glucose	39.7 ± 1.4
Fructose	37.2 ± 1.2
Insoluble fibers	8.9 ± 0.26
Skin	0.5 ± 0.15

**Table 2.** Composition of hot water-insoluble flesh fiber and skin part of palm date as well as low lignin and high lignin fibers separated from palm date fiber*.

**Component**	**Flesh fiber**	**Low lignin fiber**	**High lignin fiber**	**Skin**
Free sugar	20.6	<0.5	<0.5	3.9
Glucose	10.7 ± 1.1	<0.5	<0.5	2.1 ± 0.49
Fructose	9.9 ± 1.0	<0.5	<0.5	1.8 ± 0.48
Polysaccharide	20.9	53.1	15.8	40.8
Glucan	10.2 ± 0.25	27.2 ± 0.10	6.8 ± 0.13	15.9 ± 0.25
Xylan	4.8 ± 0.13	7.3 ± 0.03	5.4 ± 0.05	18.3 ± 0.12
Galactan	3.7 ± 0.03	13.6 ± 0.11	1.3 ± 0.07	2.7 ± 0.05
Mannan	0.5 ± 0.05	0.3 ± 0.07	0.9 ± 0.10	<0.1
Arabinan	1.7 ± 0.10	4.7 ± 0.03	1.4 ± 0.06	3.9 ± 0.04
Lignin	49.9 ± 0.25	27.2 ± 0.10	75.0 ± 0.13	41.4 ± 0.25
Acid-soluble lignin	2.2 ± 0.20	4.1 ± 0.08	3.1 ± 0.10	3.3 ± 0.09
Acid-insoluble lignin	47.7 ± 0.22	23.1 ± 0.28	71.9 ± 0.47	38.1 ± 0.27
Ash	1.6 ± 0.05	0.9 ± 0.08	1.7 ± 0.10	6.2 ± 0.04

* All data are in % of dry weight of the solid.

All the given values are means of three determinations ± standard deviation.

**Table 3.** Absorption results of FTIR spectra of high lignin and low lignin fibers, palm date fibers before and after hydrolysis.

	**TCI**	**Wave number (cm^−1^)**		
		**1518**	**1607**	**1646**
High lignin fiber	0.85 ± 0.025	0.187 ± 0.008	0.273 ± 0.010	0.160 ± 0.006
Low lignin fiber	0.71 ± 0.018	0.129 ± 0.007	0.203 ± 0.009	0.194 ± 0.008
Palm date fiber before hydrolysis	0.79 ± 0.022	0.169 ± 0.008	0.261 ± 0.011	0.188 ± 0.012
Palm date fiber after hydrolysis	1.1 ± 0.023	0.279 ± 0.012	0.318 ± 0.010	0.219 ± 0.009

All the given values are means of three determinations ± standard deviation.

**Table 4.** Yield of enzymatic hydrolysis of hemicellulose in date flesh solid part.

**Applied enzymes in enzymatic hydrolysis**	**Yield (%)^a^**

Cellulase 20FPU	37.5 ± 0.8
Cellulase 50FPU	67.1 ± 0.5
Cellulase 20FPU, β-glucosidase 50IU	81.9 ± 0.2
Cellulase 50FPU, β-glucosidase 50IU	81.4 ± 0.8
Hemicellulase	0
Cellulase 50FPU, β-glucosidase 50IU, hemicellulase 20U	84.3 ± 0.9

a (gram of glucan hydrolysed)/(gram of initial glucan) × 100.

All the given values are means of two determinations ± standard deviation.
